# Multi-functional Device with Switchable Functions of Absorption and Polarization Conversion at Terahertz Range

**DOI:** 10.1186/s11671-018-2811-z

**Published:** 2018-11-29

**Authors:** Lin Peng, Xing Jiang, Si-min Li

**Affiliations:** 1 Guangxi Key Laboratory of Wireless Wideband Communication and Signal Processing, Guilin University of Electronic Technology, Guilin, 541004 Guangxi China; 2 School of Physics, University of Electronic Science and Technology of China, Chengdu, 541004 China; 3 Guangxi University of Science and Technology, Liuzhou, 545006 Guangxi China

**Keywords:** Absorption mode, Polarization conversion mode, Terahertz, Graphene, Metasurface

## Abstract

Terahertz electromagnetic (EM) wave components usually have a single function, such as they can only convert the polarization state of an incident wave or absorb the incident energy, which would be a limitation for their applications. To make a breakthrough, a multi-functional device (MFD) is proposed in this paper, and it is capable of switching between absorption mode and polarization conversion mode. The device has a low-profile and simple structure, and it is constructed by graphene-based absorbing metasurface (AM) and gold-based polarization conversion metasurface (PCM). By controlling the chemical potential (*μ*_c_) of the graphene, the leading role is transferred between the AM and the PCM, which leads to steerable absorption and polarization conversion (PC) modes. For the PC mode, the simulated polarization conversion ratio (PCR) is larger than 0.9 in the 2.11–3.63-THz band (53.0% at 2.87 THz). For the absorption mode, the simulated absorptivity is larger than 80% in the 1.59–4.54-THz band (96.4% at 3.06 THz). The physical mechanisms and operating characteristics of the MFD are discussed. This research has potential applications in terahertz imaging, sensors, photodetectors, and modulators.

## Introduction

Absorbers and polarization converters, capable of regulating electromagnetic (EM) wave, are two crucial devices for terahertz technology. They have significant applications in sensors, photodetectors, and modulators, and they are indispensable in medical imaging/diagnostics, environmental monitoring and surveillance, chemical spectroscopy, high-resolution radar, and high-speed communication [1–4]. The absorbers are utilized to absorb and dissipate the impinging EM wave, while the polarization converters have the capacity of polarization state regulating of the illuminating wave. These devices are widely studied in recent years [4–24].

Metasurfaces are found to have perfect absorption in the terahertz wave range [5–8]. This metasurface can be constructed by gold patterns or graphene patterns. The gold patterns include coupled ring resonator and cross-shaped structure [5], cross-shaped gold resonator [6], and three-layer cross-shaped gold resonators [9]. However, the bandwidths of these gold metasurface absorbers are quite narrow. Graphene, which supports surface plasmons in the terahertz range [10, 11], is a good material to design metasurface-based absorber with a wide bandwidth. The fishnet graphene pattern achieves a bandwidth of 59.4% at 3.2 THz [12], the dual-ring structure with hybridized plasmonic resonances obtains a bandwidth of 1.18–1.64 THz (32.6%) [13], the nine layers of different size graphene ribbons realizes good absorption from 3 to 7.8 THz (88.9%) [14], and the three-layer asymmetrically pattern graphene strips etched with holes in [15] has a bandwidth of 84.6% (4.7–11.6 THz). Though the monolayer of transition metal dichalcogenides and periodic metal nano-groove array has a narrow bandwidth, it absorbs light in a wide angle [16]. In [17], monolayer MoS_2_ is applied to titanium nitride nano-disk array, which achieves an average absorption of 98.1% in the band from 400 to 850 nm (72%).

On the other hand, metasurfaces have high performance in polarization conversion. Noble metals, such as gold, have high efficiency for metasurface-based polarization converter designing. Double L-shaped pattern with two metallic gratings in [18] rotates a linear polarization (LP) by 90°. The bandwidth of the converter in [18] is 0.2–0.4 THz (66.7%). Double L-shaped pattern and grating with Fabry-Perot-like resonance achieve a bandwidth from 0.55 to 1.37 THz (85.4%) [19]. Three-layer metasurfaces form a quarter-wave converter to convert a LP incident wave to a circular polarization (CP) wave, in a bandwidth of 2.1–8 THz (116.8%) [20]. The strip-loaded half elliptical ring structure in [21] is capable of cross-polarization converting both LP and CP with a bandwidth of 2.1–2.9 THz (32%). The graphene metasurfaces applied for polarization converter usually realize the function of frequency or polarization state tuning. The designs in [22, 23] obtain polarization rotation by etching slots/hollows periodically on graphene sheets, and the operating frequencies can be dynamically tuned by adjusting the chemical potential (*μ*_c_). The periodic graphene patterns [24] and dual crossed graphene gratings [25] tune the polarization states. The design in [21] applies graphene strips on the ground to disturb the field distributions; then, the polarization conversion ratio can be regulated.

Though the above-reported absorbers and polarization converters are very efficient, these devices are a single function. They are not accommodated with terahertz systems that require portable, compact, and multi-functional devices. Therefore, multi-functional devices (MFDs) are significant. In this research, an MFD, capable of switching between absorption mode and polarization conversion mode, is proposed. The proposed MFD has a low-profile and simple structure by assembling a gold-based polarization conversion metasurface (PCM) and a graphene-based absorbing metasurface (AM). Then, by setting the chemical potential of graphene *μ*_c_ = 0 eV, the AM is neutralized and the PCM plays a dominant role, and the device rotates the polarization of an incident EM wave. By setting *μ*_*c*_ = 0.7 eV, the AM takes the main role and the device absorbs the incident EM wave.

## Methods

**Fig. 1:**
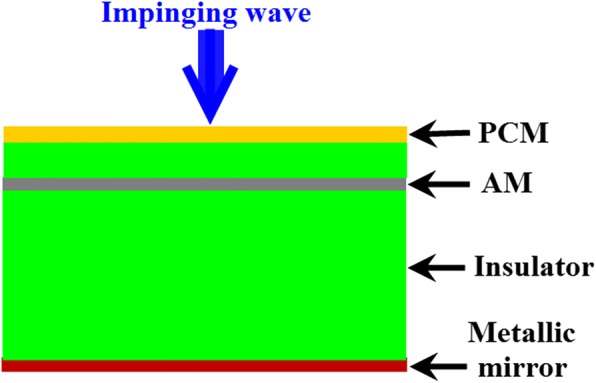
Typical configuration of a MFD

To obtain the capacity of switching between absorption and polarization conversion (PC) modes, the MFD includes two categories of metasurfaces as shown in Fig. 1. One type is absorbing metasurface (AM), and the other type is PC metasurface (PCM). A typical configuration of the MFD, as presented in Fig. 1, includes PCM structure, AM structure, metallic mirror, and insulators to separate them. It is supposed that, at the absorption mode, the AM dominates the impinging wave and dissipates the incident power, and the PCM is of no avail at this mode. At the PC mode, the AM should be neutralized and the PCM plays a leading role; therefore, the polarization state of the incident wave is converted. To attain the above claims, the key point is the neutralization of the AM at the PC mode. Therefore, the tunable material should be used to construct the AM, in which the properties of the AM can be tuned. Fortunately, the graphene demonstrates ultra-high electronic mobility and tunable conductivity by adjusting its doping level or electrical grating [26, 27]. Therefore, it is advisable to utilize graphene for AM designing. The conductivity of the graphene can be expressed by Kubo formula (1), and it includes intraband and interband contributions.

1 $$ {\displaystyle \begin{array}{l}{\sigma}_s={\sigma}_{\operatorname{int}\mathrm{ra}}\left(\omega, {\mu}_c,\varGamma, T\right)+{\sigma}_{\operatorname{int}\mathrm{er}}\left(\omega, {\mu}_c,\varGamma, T\right)\\ {}{\sigma}_{\operatorname{int}\mathrm{ra}}\left(\omega, {\mu}_c,\varGamma, T\right)=-j\frac{e^2{k}_BT}{\pi {\mathrm{\hslash}}^2\left(\omega -j2\Gamma \right)}\left(\frac{\mu_c}{k_BT}+2\ln \left({e}^{-\frac{\mu_c}{k_BT}}+1\right)\right)\\ {}{\sigma}_{\operatorname{int}\mathrm{er}}\left(\omega, {\mu}_c,\varGamma, T\right)\cong -j\frac{e^2}{4\pi \mathrm{\hslash}}\ln \left(\frac{2\left|{\mu}_c\right|-\left(\omega -j2\Gamma \right)\mathrm{\hslash}}{2\left|{\mu}_c\right|+\left(\omega -j2\Gamma \right)\mathrm{\hslash}\Big)}\right)\end{array}} $$

where *e*, ℏ, *k*_*B*_, *T*, and *μ*_c_ represent the charge of an electron, the reduced Planck’s constant, Boltzmann’s constant, Kelvin’s temperature, and chemical potential, respectively. The *Γ* is a phenomenological scattering rate, and it is assumed to be independent of energy *ε*. Thus, the complex conductivity *σ*_*s*_ can be adjusted by tuning the chemical potential (*μ*_c_) with biasing voltage. It is found in Eq. (1) that for *μ*_c_ = 0 eV, the conductivity of the graphene is very small owing to the low carrier density at this case. Therefore, the graphene operates as a dielectric substrate. Moreover, as the graphene layer is very thin, it has little impact on the illuminated EM waves for *μ*_c_ = 0 eV. However, the carrier density of the graphene would be raised with increasing chemical potential (*μ*_c_), and the complex conductivity (*σ*_*s*_) of the graphene is boosted with increasing chemical potential (*μ*_c_) [26, 27]. Therefore, the graphene supports surface plasmon polaritons (SPPs) for large *μ*_c_ [26, 28–30], and the SPPs confine the incident waves. To further enhance the SPPs and achieve wave absorption in certain frequencies, periodical structures should be etched in the graphene layer to form a metasurface, which is called AM. Therefore, by setting *μ*_c_ = 0, the AM can be deemed as a thin dielectric substrate, and it is almost transparent to EM wave. Thereby, the incident EM wave can be concentrated on the PCM layer, and the device operates in the PC mode. For an appropriate large *μ*_c_, the enhanced SPPs of the AM confine most of the incident EM wave, which makes the PCM layer of no avail. Thereby, the incident EM waves are dissipated in the AM layer.

**Fig. 2:**
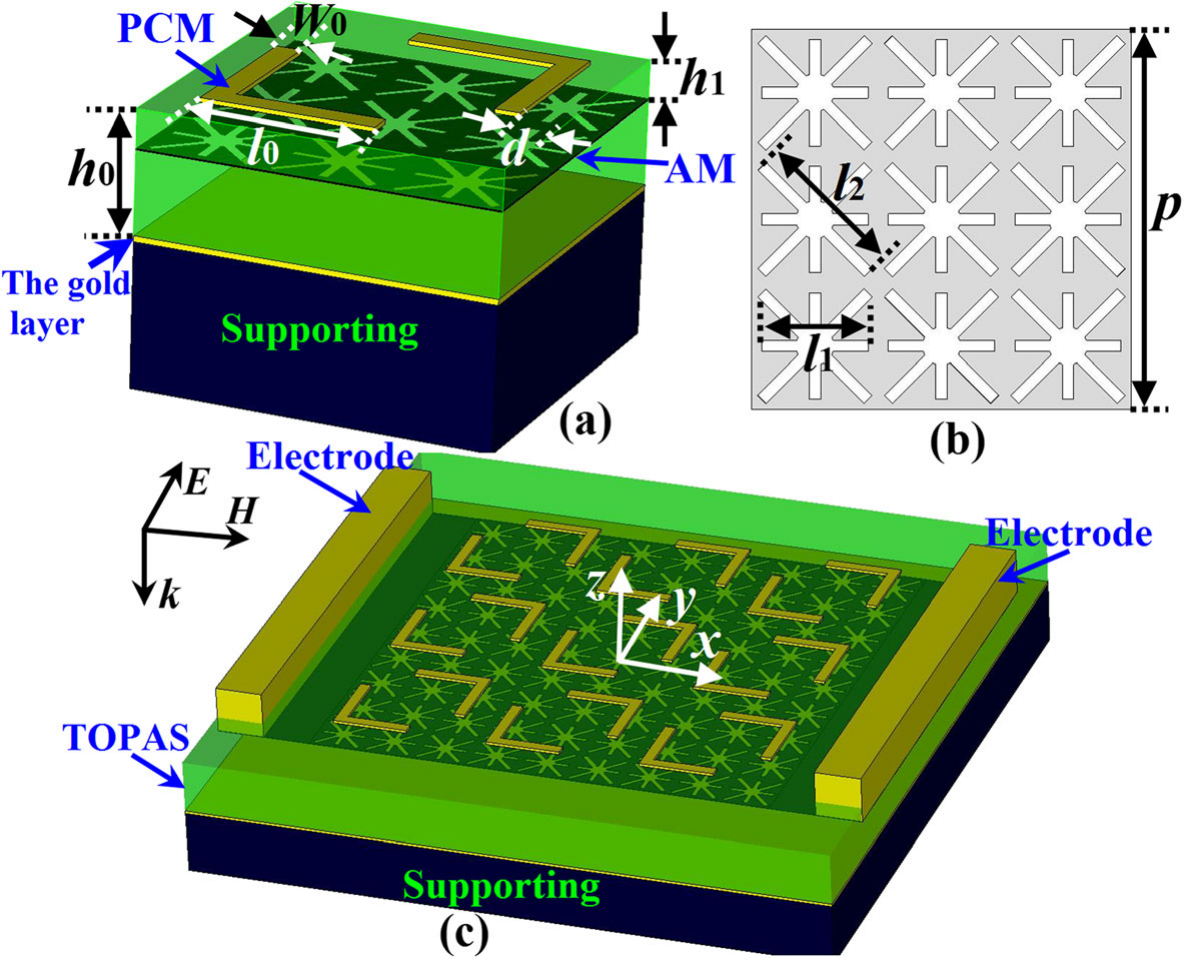
Schematic view of the proposed MFD with absorbance and polarization conversion modes. **a** 3D view of a cell. **b** Top view of the graphene AM in one cell. **c** 3D view of the array

According to the above discussion, a low-profile MFD with gold-based PCM and graphene-based AM is proposed as shown in Fig. 2. Figure 2a is a 3D view of a cell. It is found in the figure that one layer of gold-based PCM is printed on the top of the substrate TOPAS polymer [31]. The PCM pattern is a dual L-shaped structure with wide band and good polarization conversion characteristics [18, 19]. As demonstrated in Fig. 2a, a graphene-based AM is inserted in the TOPAS polymer substrate with a distance *h*_1_ to the PCM. To endow the graphene-based AM a dominant role at the absorption mode, the AM should have strong SPPs at a certain chemical potential (*μ*_c_) to confine most of the incident power and neutralize the PCM. For this purpose, patterns of cross-slots are etched in a graphene layer, as exhibited in Fig. 2b. It is supposed that the cross-slot patterns bring periodic changing (*σ* = 0) to the uniformity complex conductivity of the graphene, which leads to charge density rearranging and focusing. Therefore, SPPs are generated and enhanced. The cross-slot structure, as demonstrated in Fig. 2b, is capable of concentrating carrier and fields around the slots, which ensures strong SPPs. The slot lengths of *l*_1_ and *l*_2_ are choosing to ensure the resonances of the AM fall into operating scope of the PCM; therefore, one cell of the AM has 3 × 3 cross-slot patterns. Note that the PCM and AM are moving and operating independently as their on-off is controlled by the chemical potential (*μ*_c_); therefore, the PCM pattern and the AM pattern could be other architectures. The TOPAS polymer is an excellent substrate material for broadband terahertz design, and its refraction index is approximately 1.53 with a very low loss. A gold layer is printed at the bottom of the TOPAS polymer substrate for total reflection. The gold layer is supported by a substrate, which can be Si. The thickness of the gold is 200 nm. Note that the supporting material has no effect on the performances on the MFD as there are no impinging waves penetrating the gold layer. As demonstrated from a 3D view of the array in Fig. 2c, the chemical potential can be adjusted through biasing the voltage. The MFD can be fabricated by repeating the growth and transfer process [32, 33]. The graphene AM is supposed to have *T* = 300 K and momentum relaxation time *τ* = 0.1 ps. For PC mode, *μ*_c_ = 0 eV. The chemical potential for absorption mode is *μ*_c_ = 0.7 eV. The optimized parameters of the MFD are *h*_0_ = 17 μm, *h*_1_ = 1.5 μm, *l*_0_ = 24 μm, *W*_0_ = 2 μm, *l*_1_ = 14 μm, *l*_2_ = 19.8 μm, and *p* = 50 μm.

## Results, Physical Mechanisms, and Discussion

### Results

**Fig. 3:**
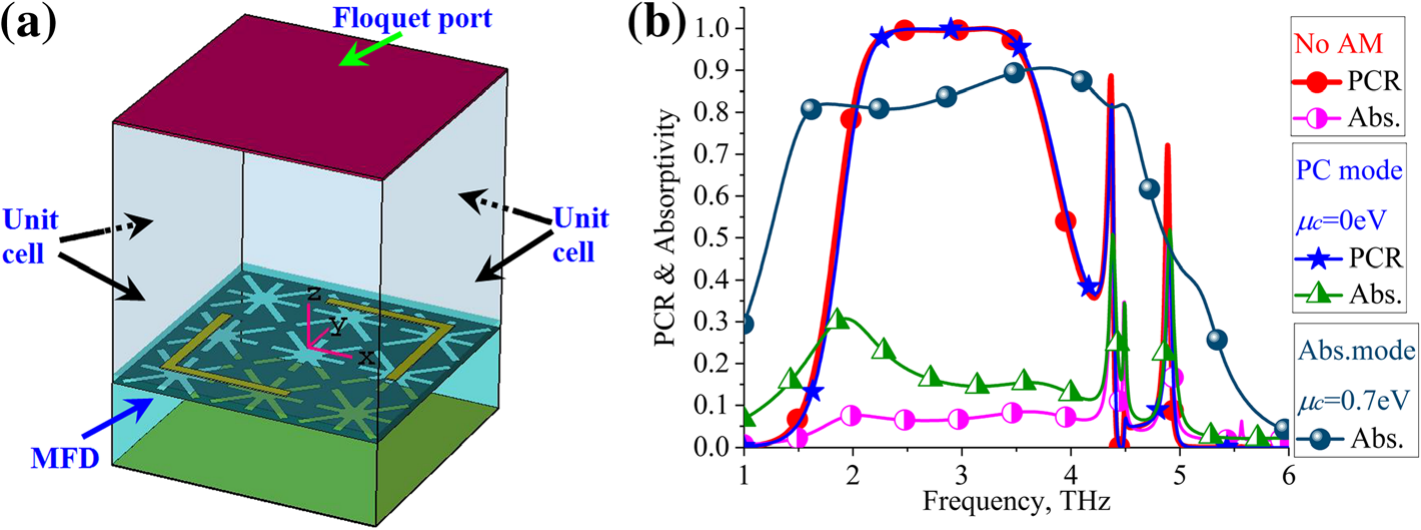
PCR and absorptivity calculation of the proposed MFD. **a** Simulation model. **b** Calculated results of the PC mode and absorption mode; the results of the structure without AM are also demonstrated for comparison. **b** The PCR and absorptivity of the structure without AM are plotted as the red curve with solid circle mark and carmine curve with semi-solid circle mark, respectively. For the PC mode of the proposed MFD, the PCR and absorptivity are plotted as the blue curve with solid five-pointed star mark and cyan curve with semi-solid delta mark, respectively. For the absorption mode of the proposed MFD, the absorptivity is plotted as the hidden blue curve with full sphere mark

The proposed MFD was simulated, and the polarization conversion ratio (PCR) and the absorptivity of the proposed MFD were calculated. As shown in Fig. 3a, the full-wave analyses are conducted in CST Studio Suite with frequency domain solver. Therefore, unit cell boundaries are set at the periphery sides, and a floquet port is set at the top of the calculation region. The PCR and absorptivity of the structure without AM are also plotted in the figure for comparison. Note that the PCR and absorptivity can be calculated through the reflection coefficients of the structure since there is no transmission owing to the gold layer [34]. Here, the terms are explicitly defined according to *y*-polarized illumination. The electric field of the *y*-polarized incidence wave is defined as *E*_*iy*_, and the reflected wave includes a *y*-polarized electric field (*E*_*ry*_) and *x*-polarized electric fled (*E*_*rx*_). Then, the reflection coefficients of co-polarization and cross-polarization are defined as *r*_*yy*_ = *E*_*ry*_/*E*_*iy*_ and *r*_*xy*_ = *E*_*rx*_/*E*_*iy*_, respectively. Therefore, the PCR and absorptivity can be calculated by Eqs. (2) and (3), respectively. Note that the PCR and absorptivity of *x*-polarized incidence can be analogously calculated according to Eqs. (2) and (3).

2 $$ \mathrm{PCR}={r^2}_{xy}/\left({r^2}_{yy}+{r^2}_{xy}\right) $$

3 $$ \mathrm{Abs}.=1-{r^2}_{yy}-{r^2}_{xy} $$

As shown in Fig. 3b, the MFD operates at PC mode with *μ*_c_ = 0 eV, and it works at absorption mode with *μ*_c_ = 0.7 eV. At the PC mode, the structure operates as a polarization converter, and it rotates a linear polarized incident wave to its orthogonal polarization wave. For the PC mode, the PCR is larger than 0.9 in the 2.11–3.63-THz band (53.0% at 2.87 THz), while the absorptivity is small and it ranges from 0.14 to 0.27 in the band. For the structure without AM, it has almost the same PCR band as the PC mode while its absorptivity ranges from 0.06 to 0.09. In the absorption mode, most of the incident wave is absorbed in the band as demonstrated in the figure. Note that the PCR curve for absorption mode is not presented as it is meaningless. The absorptivity is larger than 80% in the 1.59–4.54-THz band (96.4% at 3.06 THz). Therefore, by adjusting the chemical potential, the proposed structure can switch between PC mode and absorption mode.

### Physical Mechanisms

**Fig. 4:**
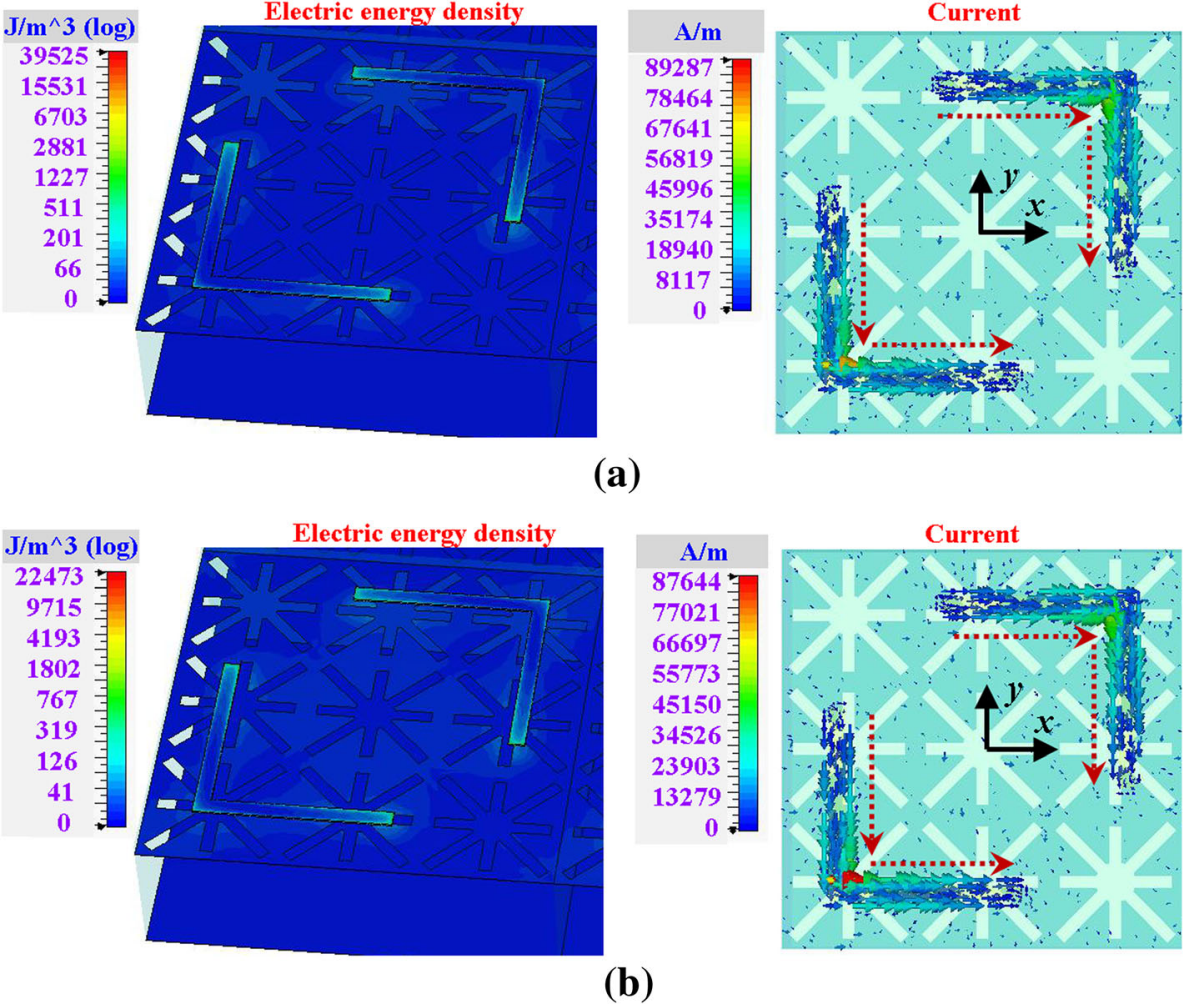
Field distributions of the PC mode (*μ*_c_ = 0 eV). **a** 2.56 THz. **b** 3.22 THz

**Fig. 5:**
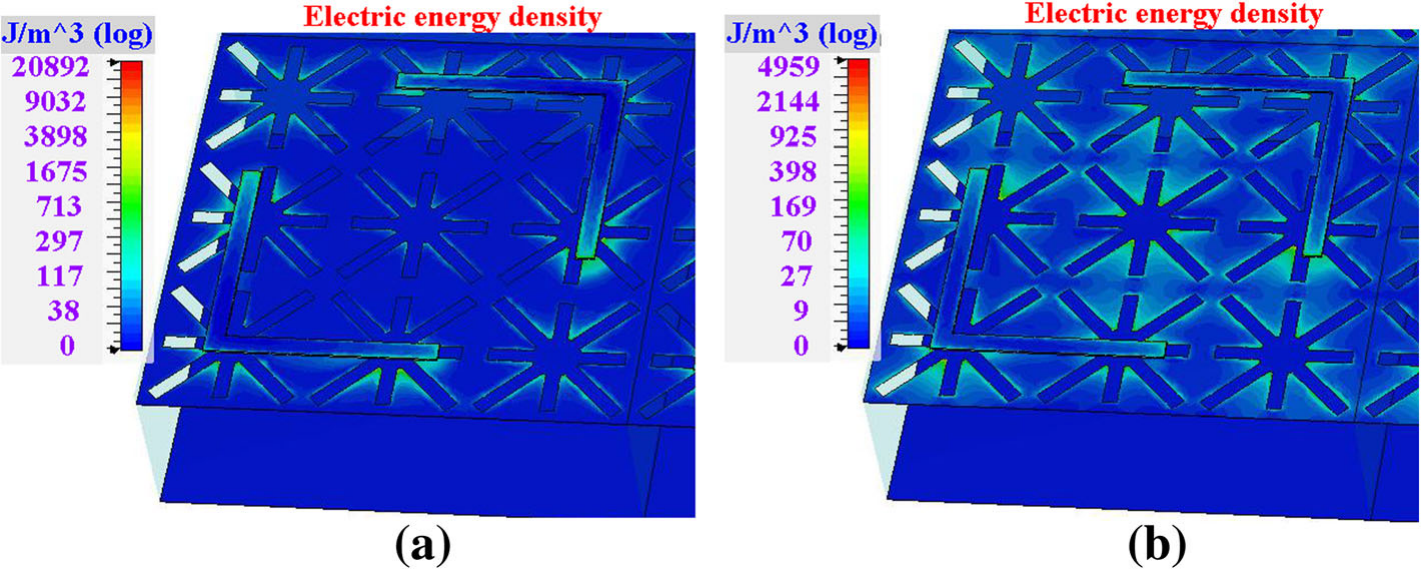
Field distributions of the absorption mode (*μ*_c_ = 0.7 eV). **a** 1.7 THz. **b** 3.3 THz

To further reveal the physical mechanisms of the switching characteristics of the two modes, the electric energy densities at the PC mode and the absorption mode of the structure are presented in Figs. 4 and 5, respectively. The current distributions of the PC mode are also plotted in Fig. 4 to state the polarization conversion characteristic. The current distributions of the absorption mode are not illustrated as the currents are attenuated and dissipated at this mode. Note that the field distributions are obtained under *y*-polarized illuminations.

For the PC mode (*μ*_c_ = 0 eV), two frequencies of 2.56 THz and 3.22 THz are chosen to present their field distributions at Fig. 4a and b, respectively. The left parts of the figures are the electric energy densities, and the right parts are the currents. As shown in the figures, the field distributions of 2.56 THz and 3.22 THz are very similar to each other, which imply a wide operating band. From the electric energy densities at the left parts of Fig. 4a, b, the energies are mainly concentrated on the L-shaped structures (PCM). It is indicated that the PCM plays a leading role for *μ*_c_ = 0 eV. From the currents at the right parts of Fig. 4a, b, the currents of both 2.56 THz and 3.22 THz are also concentrated on the PCM, and the currents on the AM are weak. The dotted line arrows indicate the vectors of the currents. The *y*-polarized illuminations generate *x*-vector currents on the L-shaped structures, which achieve polarization conversion.

For the absorption mode (*μ*_c_ = 0.7 eV), the electric energy densities of 1.7 THz and 3.3 THz are painted in Fig. 5a and b, respectively. As shown in the figure, the electric energy densities of the two frequencies are mainly distributed on the AM. It is also found that the energies are focused in the cross-slot patterns; therefore, SPP effects are enhanced by the cross-slots on the AM. The strong SPP effects lead to field enhancement on the AM, which endow the AM a dominant role. Thereby, the incident waves are confined and dissipated in the AM. It is also found that there are still some energies spread on the PCM, which make no perfect absorption, such as 80–90% absorptivity in the band.

## Discussion

**Fig. 6:**
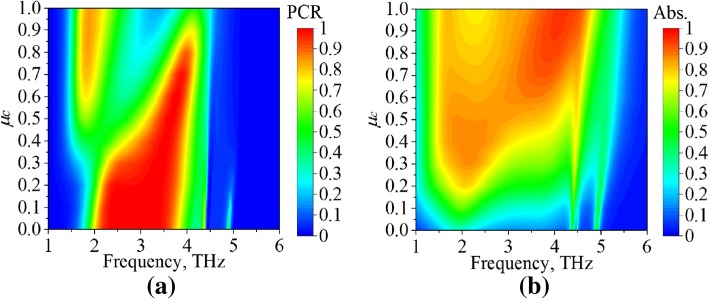
Characteristics of the proposed MFD for different chemical potentials (*μ*_*c*_). **a** PCR. **b** Absorption

To further reveal the characteristics of the proposed MFD, parametric studies are discussed in this section. Figure 6a and b present the PCR and absorption characteristics, respectively, in terms of the chemical potential (*μ*_c_). As shown in Fig. 6a, a smaller *μ*_c_ means smaller conductivity of the AM, and the PCM has a stronger role. Therefore, good PCR is observed with *μ*_c_ = 0 eV, and it is deteriorated with increasing *μ*_c_. The absorption characteristic of the MFD presents almost contrary tendency as shown in Fig. 6b. With *μ*_c_ increased from 0 to 1 eV, the SPPs on the AM are inspired and enhanced. Thus, the incident EM waves are confined in the AM, and the power is absorbed. The *μ*_c_ = 0.7 eV is chosen for the widest bandwidth. It is also noticed in Fig. 6a that the PCR values around 1.85 THz are larger than 80% for 0.7 eV < *μ*_c_ < 1 eV; however, most of the powers are dissipated for these *μ*_c_s as indicated in Fig. 6b. Therefore, the chemical potential (*μ*_c_) is a valuable parameter to adjust the PCR and absorption characteristics.

**Fig. 7:**
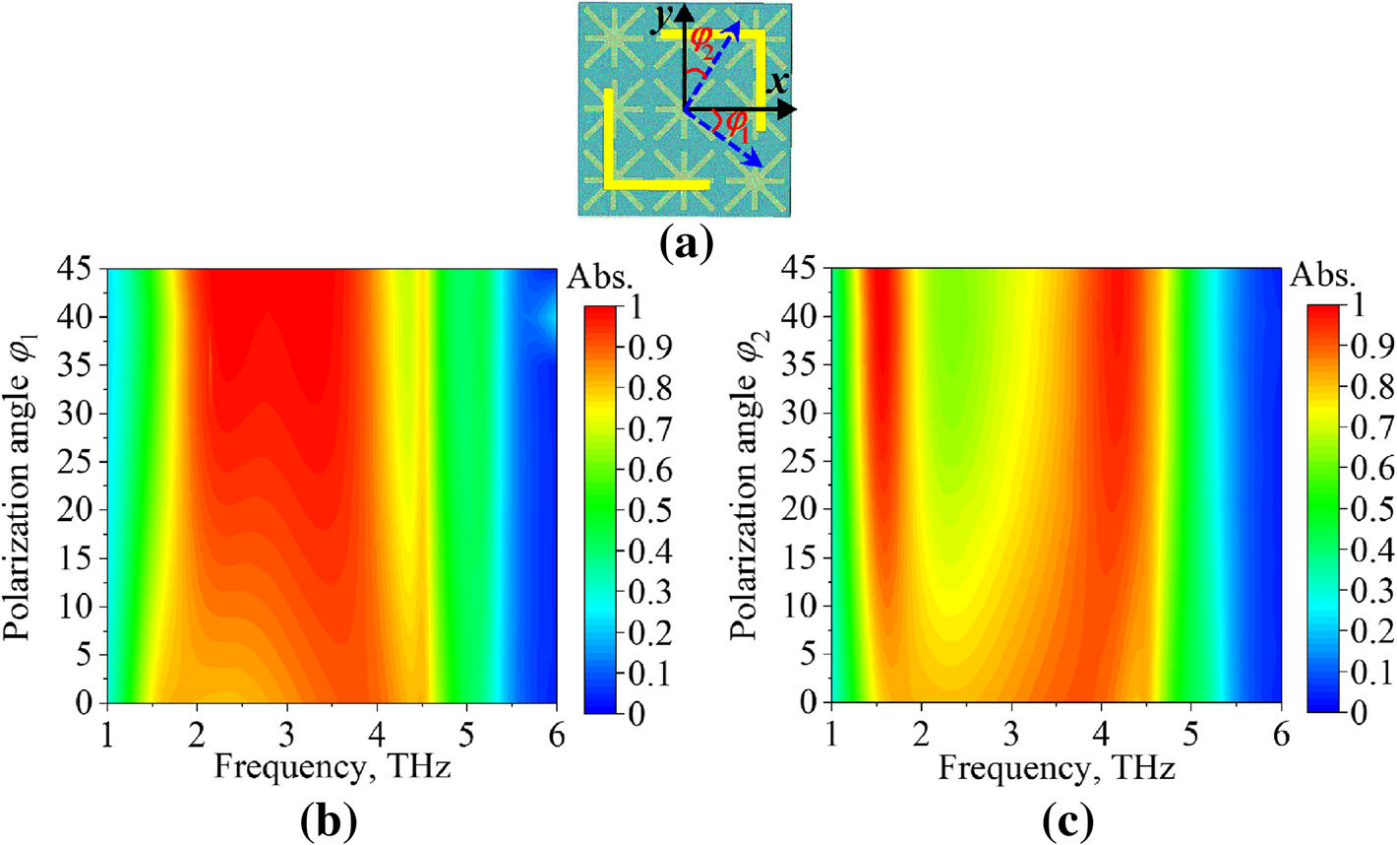
The absorption characteristics of the absorption mode (*μ*_c_ = 0.7 eV) under normal incidence for different polarization angles (*φ*). **a** The *φ*_1_ and *φ*_2_ are the angle of the incident electric field relative to *x-* and *y*-axes, respectively. **b** *φ*_1_. **c** *φ*_2_

The absorptivity of the absorption mode for different polarization angles (*φ*_1_ and *φ*_2_) is depicted in Fig. 7. As depicted in Fig. 7a, the *φ*_1_ and *φ*_2_ are the angles of the incident electric fields relative to *x*- and *y*-axes, respectively. According to the symmetric structure of the MFD, the *φ*_1_ and *φ*_2_ varied from 0 to 45°. In Fig. 7b, as the *φ*_1_ increased from 0 to 45°, the absorptivity in the band increased from 0.8 to nearly 1, though the band is narrowed a bit with increasing *φ*_1_. As exhibited in Fig. 7c, the increasing of *φ*_2_ debases the absorptivity around 2–3 THz, and two absorption bands are obtained around 1.7 THz and 4 THz.

**Fig. 8:**
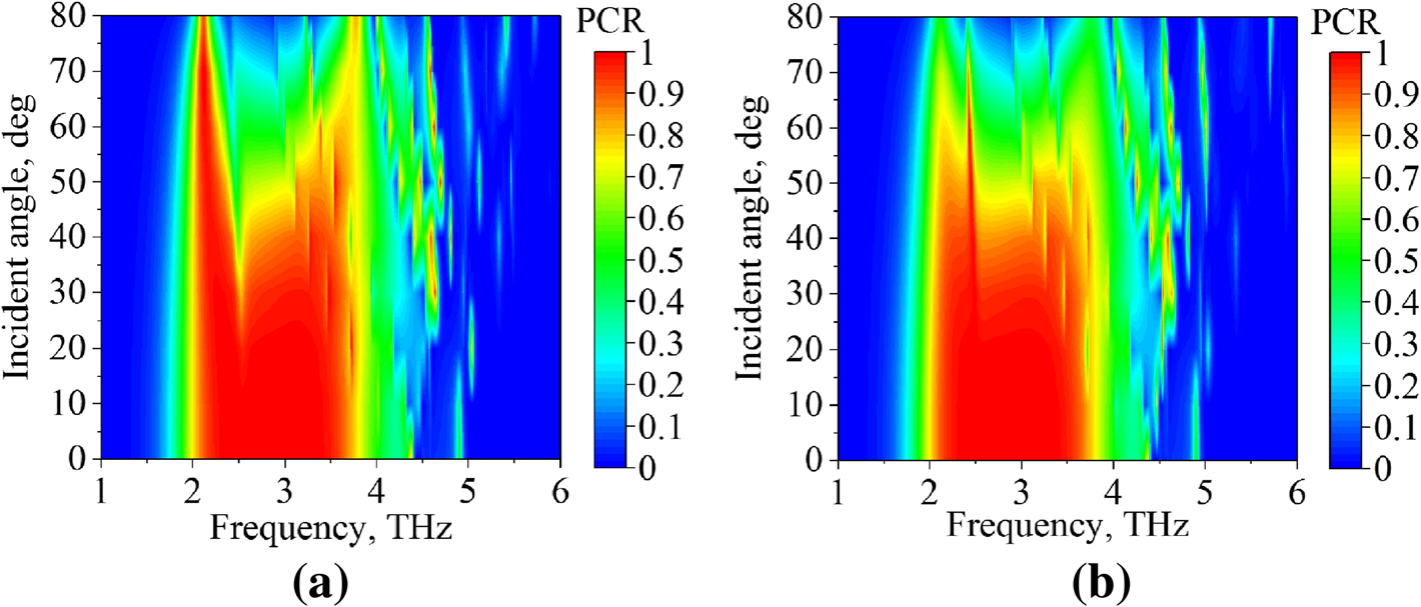
The PCR characteristics of PC mode (*μ*_*c*_ = 0 eV) for different incident angles, illuminated by **a** *s*-polarized and **b** *p*-polarized waves

**Fig. 9:**
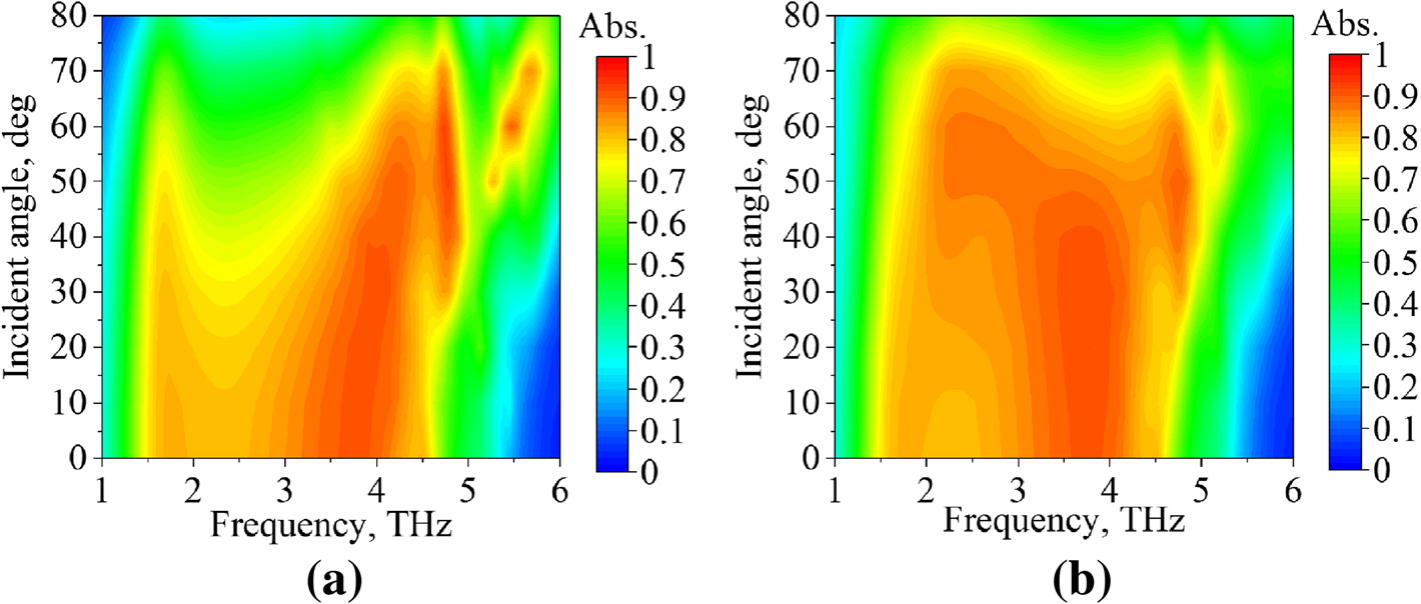
The absorption characteristics of absorption mode (*μ*_c_ = 0.7 eV) for different incident angles, illuminated by **a** *s*-polarized and **b** *p*-polarized waves

The performances of PC mode and absorption mode in terms of incident angle (*θ*) are presented in Figs. 8 and 9, respectively. Figure 8a and b demonstrate the PCR plots of *s*- and *p*-polarized incident waves, respectively, with the incident angle ranged from 0 to 80°. As shown in the figures, the PCR deteriorated with increasing *θ*; however, good PCR characteristic is also obtained for *θ* smaller than 40°. The PCR bandwidth is stable to the incident angle (*θ*). It is also found that the PCR performance of *s*-polarized incidence is insensitive to the incident angle (*θ*) for the frequencies around 2.1 THz.

For the absorption mode, the absorptivity plots of *s*- and *p*-polarized incident waves are plotted in Fig. 9a and b, respectively, with the incident angle (*θ*) ranged from 0 to 80°. Generally speaking, the absorptivity of the *s*-polarized incidence reduced with increasing *θ*, and the absorptivity is larger than 0.8 for *θ* smaller than 30°. It is interesting to find that the absorptivity of *p*-polarized incident EM wave increased with increasing *θ*.

**Fig. 10:**
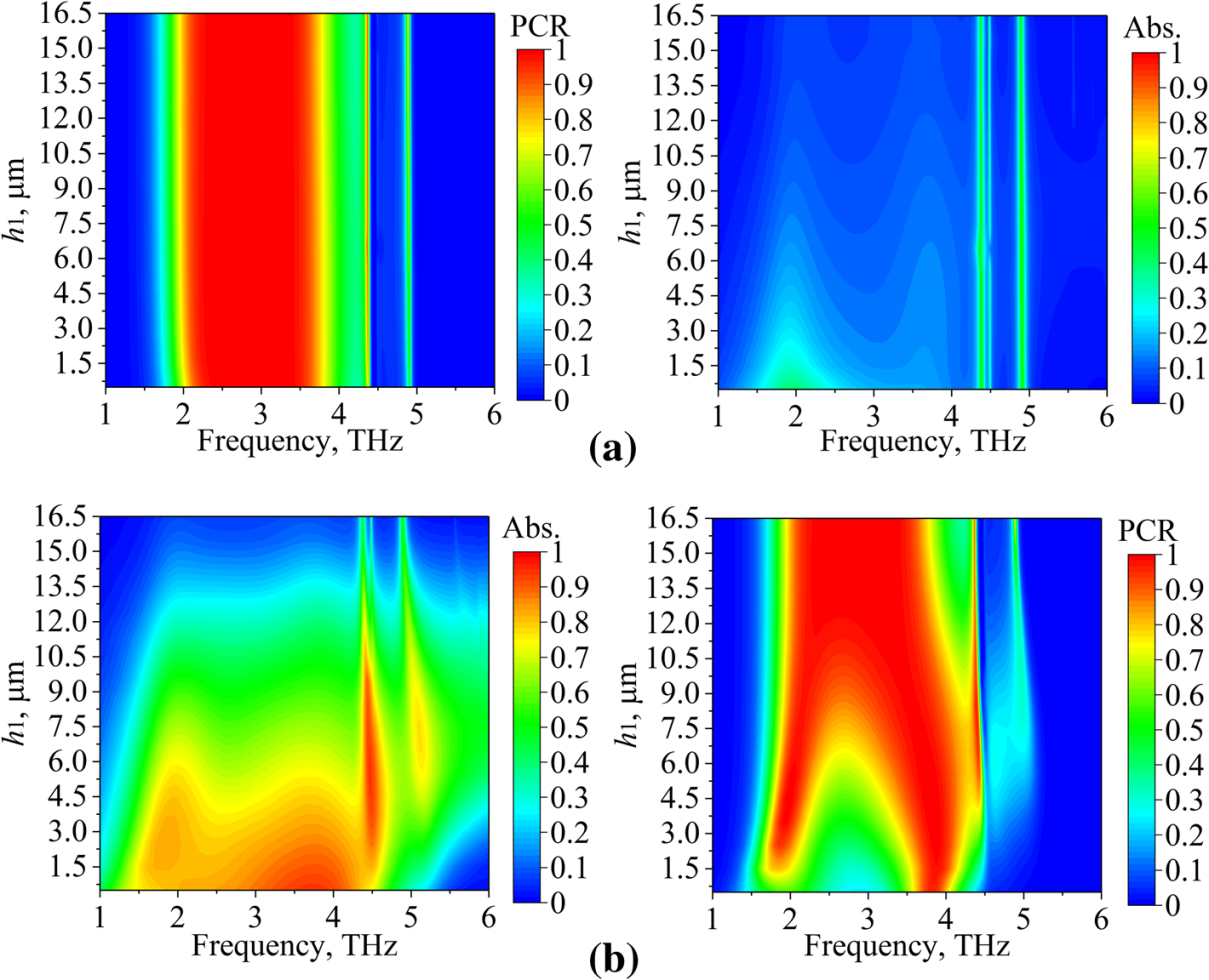
The PCR and absorption in terms of *h*_1_. **a** PC mode (*μ*_c_ = 0 eV). **b** Absorption mode (*μ*_c_ = 0.7 eV)

The structure parameter *h*_1_ is also studied to further reveal the multiple functions of the device. As the *h*_1_ is adjusted, the position of the AM is changed. Note that other structure parameters are not discussed here for simplicity. Figure 10a and b demonstrate the results of the PC mode and absorption mode, respectively. As shown in the left part of Fig. 10a, at PC mode, the *h*_1_ has little impact on the PCR. In the right part of Fig. 10b, the absorptions are also stable for *h*_1_ ranging from 0.5 to 16.5 μm, though smaller *h*_1_ has larger absorption. The results in Fig. 10a verify the discussions in the “Methods” section, and the AM is operated as thin substrate at PC mode (*μ*_c_ = 0 eV). For the absorption mode (*μ*_c_ = 0.7 eV), the AM plays a leading role; therefore, the *h*_1_ is important at this mode. As shown in the left part of Fig. 10a, the increasing of *h*_1_ decrease the absorptivity. It is because the multiple reflections and superpositions between the AM and the gold layer are important to inspire the SPPs and enhance the fields on the AM [35]. In the right part of Fig. 10b, good PCR is observed for larger *h*_1_. Therefore, in the designing of the MFD, the parameter *h*_1_ can be only considered in the absorption mode as it has little impact on the PC mode.

## Conclusions

In summary, a low-profile and simple structure MFD is proposed by combining gold-based PCM and graphene-based AM. The chemical potential (*μ*_c_) can be utilized to activate or neutralize the graphene-based AM, and then, the structure can be transformed from absorber to polarization converter. For the PC mode, the PCR is larger than 0.9 in the 2.11–3.63-THz band (53.0% at 2.87 THz). For the absorption mode, the absorptivity is larger than 80% in the 1.59–4.54-THz band (96.4% at 3.06 THz). The design may be applied to terahertz imaging, sensing, photodetection, and modulation systems.

## Abbreviations

AM: Absorbing metasurface
CP: Circular polarization
EM: Electromagnetic
LP: Linear polarization
MFD: Multi-functional device
PC: Polarization conversion
PCM: Polarization conversion metasurface
PCR: Polarization conversion ratio
SPPs: Surface plasmon polaritons
